# Supplementary material for: The Small RNA Universe of Capitella teleta
Source: Front Mol Biosci. 2022 Feb 25;9:802814. doi: 10.3389/fmolb.2022.802814 (PMC8915122; doi:10.3389/fmolb.2022.802814)
Supplement: Supplementary file 1 [file DataSheet1.ZIP › Supplement/known/CAPTEscaffold_192_13677.pdf]

Provisional ID	:	CAPTEscaffold_192_13677
Score total	:	134820.4
Score for star read(s)	:	3.9
Score for read counts	:	134810.6
Score for mfe	:	1.2
Score for randfold	:	1.6
Score for cons. seed	:	3
Total read count	:	264437
Mature read count	:	259365
Loop read count	:	0
Star read count	:	5072

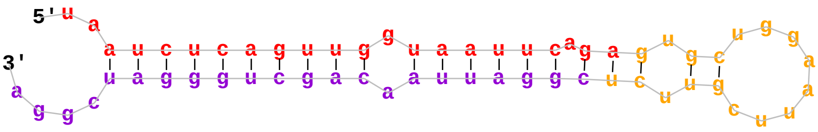

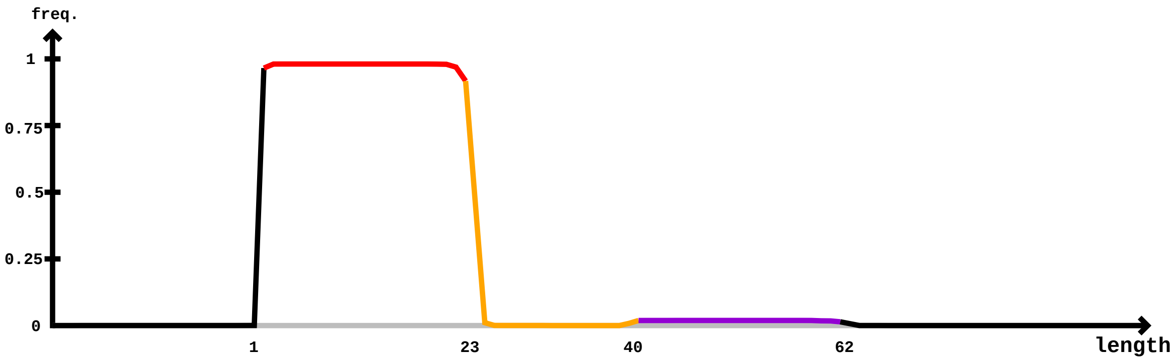

Mature **Star**

```
5'- ccuccuagugacuaauugucuaaucucaguugguaauucagagugcuggaauucguucucggauuaacagcugggaucggacaaauaacacacuaacaucuacaccacaaaa  -3'  obs
    ccuccuagugacuaauugucuaaucucaguugguaauucagagugcuggaauucguucucggauuaacagcugggaucggacaaauaacacacuaacaucuacaccacaaaa        exp
    ccuccuagugacuaauugucuaaucucaguugguaauucagagugcuggaauucguucucggauuaacagcugggaucggacaaauaacacacuaacaucuacaccacaaaa        known
    .....(((((..((.((((((.((((((((((.((((((.(((.((........)).))))))))).)))))))))).)))))).))...))))).................  reads  mm  sample

    ccuccuagugacuaauuguc............................................................................................  11  0  seq
    ccuccuagugacuaauugucu...........................................................................................  1  0  seq
    .cuccuagugacuaauuguc............................................................................................  13  0  seq
    ..uccuagugacuaauuguc............................................................................................  4  0  seq
    ................ugucuaaucucaguugguaauu..........................................................................  3  0  seq
    ..................ucuaGucucaguugguaauu..........................................................................  3  1  seq
    ..................ucuaaucucaguugguaauuc.........................................................................  6  0  seq
    ..................ucuaaucucaguugguaauucaga......................................................................  3  0  seq
    ...................Auaaucucaguugguaauucag.......................................................................  4  1  seq
    ...................Auaaucucaguugguaauucaga......................................................................  6  1  seq
    ...................cuaaucucaguuggAaauucaga......................................................................  1  1  seq
    ...................cuaaucucaguugguaauucaga......................................................................  21  0  seq
    ...................cuaaucucaguugguaauucagag.....................................................................  5  0  seq
    ...................Auaaucucaguugguaauucagag.....................................................................  2  1  seq
    ....................Aaaucucaguugguaauu..........................................................................  1  1  seq
    ....................uaGucucaguugguaauu..........................................................................  1  1  seq
    ....................uaaucucaguugguaauu..........................................................................  74  0  seq
    ....................uaaucucaguugguaauuU.........................................................................  1  1  seq
    ....................uaaucucaguugguaauuc.........................................................................  163  0  seq
    ....................uaaucucagGugguaauuc.........................................................................  1  1  seq
    ....................uaaucCcaguugguaauuc.........................................................................  1  1  seq
    ....................uaaucucaguugguaauuAa........................................................................  4  1  seq
    ....................Gaaucucaguugguaauuca........................................................................  4  1  seq
    ....................uaaucucaguugguaauucU........................................................................  4  1  seq
    ....................uaaucucaguugguaaAuca........................................................................  2  1  seq
    ....................uaaucucaguugguaauAca........................................................................  1  1  seq
    ....................uaaucAcaguugguaauuca........................................................................  2  1  seq
    ....................Aaaucucaguugguaauuca........................................................................  11  1  seq
    ....................uaauAucaguugguaauuca........................................................................  2  1  seq
    ....................uaGucucaguugguaauuca........................................................................  23  1  seq
    ....................uaaucucaguugguaauucG........................................................................  2  1  seq
```

```
                    Mature                                       Star
ccuccuagugacuaauugucuaaucucaguugguaauucagagugcuggaauucguucucggauuaacagcugggaucggacaaauaacacacuaacaucuacaccacaaaa
....................uaaucucaguuggAaauuca........................................................................    1       1    seq
....................uaaucucagAugguaauuca........................................................................    3       1    seq
....................uaaucucaguAgguaauuca........................................................................    1       1    seq
....................uaaucucGguugguaauuca........................................................................    2       1    seq
....................uaaGcucaguugguaauuca........................................................................    1       1    seq
....................uaaAcucaguugguaauuca........................................................................    2       1    seq
....................uaaucucaguuAguaauuca........................................................................    2       1    seq
....................uaaucucaguugguaauuca........................................................................    2739    0    seq
....................uaaucuAaguugguaauuca........................................................................    1       1    seq
....................uaaucucaguugguGauuca........................................................................    2       1    seq
....................uaaucucaguugguaauuUa........................................................................    1       1    seq
....................uaaucucaguugUuaauuca........................................................................    1       1    seq
....................uaaucucaguugguaauucUg.......................................................................    2       1    seq
....................uaaucucaguugguaauuUag.......................................................................    11      1    seq
....................uaaAcucaguugguaauucag.......................................................................    7       1    seq
....................uaauAucaguugguaauucag.......................................................................    2       1    seq
....................uaaucucaguugguaauGcag.......................................................................    1       1    seq
....................Gaaucucaguugguaauucag.......................................................................    5       1    seq
....................uaaucucaCuugguaauucag.......................................................................    1       1    seq
....................uaaucucUguugguaauucag.......................................................................    2       1    seq
....................uaaucuAaguugguaauucag.......................................................................    7       1    seq
....................uaaucucaguugguaauAcag.......................................................................    4       1    seq
....................uaaucucaguugguaauucaU.......................................................................    6       1    seq
....................uaaucucaguugguaauucaC.......................................................................    2       1    seq
....................uaaucucaguugguaauuAag.......................................................................    5       1    seq
....................uaaucGcaguugguaauucag.......................................................................    1       1    seq
....................uaaucucaguuggAaauucag.......................................................................    3       1    seq
....................uaaucucaguuUguaauucag.......................................................................    2       1    seq
....................uaaucucaguugguaauucGg.......................................................................    6       1    seq
....................uGaucucaguugguaauucag.......................................................................    2       1    seq
....................uaaucucaguugguaaGucag.......................................................................    1       1    seq
....................uaaucucaguugCuaauucag.......................................................................    1       1    seq
....................uaaucAcaguugguaauucag.......................................................................    6       1    seq
....................uaaucucaguuCguaauucag.......................................................................    2       1    seq
....................uaaucucaguAgguaauucag.......................................................................    6       1    seq
....................Aaaucucaguugguaauucag.......................................................................    58      1    seq
....................uaaucucaguugAuaauucag.......................................................................    2       1    seq
....................uaaCcucaguugguaauucag.......................................................................    4       1    seq
....................uaaucucaguugUuaauucag.......................................................................    1       1    seq
....................uaaucucaguCgguaauucag.......................................................................    1       1    seq
....................uaaucucaguuAguaauucag.......................................................................    15      1    seq
....................uaaucucaUuugguaauucag.......................................................................    1       1    seq
....................uaaucuUaguugguaauucag.......................................................................    9       1    seq
....................uaaucucaguugguaauucaA.......................................................................    22      1    seq
....................uaaucucaguugguGauucag.......................................................................    2       1    seq
....................uaaucucaguugguaaAucag.......................................................................    4       1    seq
....................uaaucucaguugguaauucag.......................................................................    13319   0    seq
....................uaauUucaguugguaauucag.......................................................................    3       1    seq
....................uaaucucagAugguaauucag.......................................................................    5       1    seq
....................uaaucucGguugguaauucag.......................................................................    5       1    seq
....................uaaucucaguugguaauuGag.......................................................................    2       1    seq
....................Naaucucaguugguaauucag.......................................................................    5       1    seq
....................uaaucucaguuggCaauucag.......................................................................    3       1    seq
....................uaaucucaguugguaUuucag.......................................................................    1       1    seq
....................uaaucuGaguugguaauucag.......................................................................    2       1    seq
....................uaaucucagCugguaauucag.......................................................................    2       1    seq
....................uaaucucaguugguaauCcag.......................................................................    2       1    seq
....................uaGucucaguugguaauucag.......................................................................    107     1    seq
....................Caaucucaguugguaauucaga......................................................................    19      1    seq
....................uaaucucaguugguaCuucaga......................................................................    2       1    seq
....................uaCucucaguugguaauucaga......................................................................    1       1    seq
....................uaaucucaguugguUauucaga......................................................................    8       1    seq
....................uaaucucagCugguaauucaga......................................................................    37      1    seq
....................uaaucucaguuAguaauucaga......................................................................    167     1    seq
....................uaaucuGaguugguaauucaga......................................................................    25      1    seq
....................uaauGucaguugguaauucaga......................................................................    4       1    seq
....................uaaCcucaguugguaauucaga......................................................................    17      1    seq
....................uaaucucagAugguaauucaga......................................................................    90      1    seq
....................uaaucucUguugguaauucaga......................................................................    17      1    seq
....................uaaucucaguugguaauucagC......................................................................    3       1    seq
```

```
                    Mature                                        Star
ccuccuagugacuaauugucuaaucucaguugguaauucagagugcuggaauucguucucggauuaacagcugggaucggacaaauaacacacuaacaucuacaccacaaaa
....................uaaucucaguugguaauucaUa......................................................................    17      1   seq
....................uaaucucGguugguaauucaga......................................................................    23      1   seq
....................uaauAucaguugguaauucaga......................................................................    21      1   seq
....................uaaucuAaguugguaauucaga......................................................................    79      1   seq
....................uaaucucaguCgguaauucaga......................................................................    28      1   seq
....................uaaucucaguugUuaauucaga......................................................................    11      1   seq
....................uaaucucaguugguaauuUaga......................................................................    125     1   seq
....................uaaucucaguugguaauAcaga......................................................................    50      1   seq
....................uaaucucaNuugguaauucaga......................................................................    1       1   seq
....................uaaucucaguugguGauucaga......................................................................    64      1   seq
....................uaaucNcaguugguaauucaga......................................................................    4       1   seq
....................uaaucucaguugguaGuucaga......................................................................    27      1   seq
....................uaaucucaguuggAaauucaga......................................................................    36      1   seq
....................uaaucucaguugguaaCucaga......................................................................    19      1   seq
....................uaaucucaAuugguaauucaga......................................................................    6       1   seq
....................uaaucucaguugguaauucaga......................................................................    155078  0   seq
....................uaaucucaguuggCaauucaga......................................................................    29      1   seq
....................uaaucGcaguugguaauucaga......................................................................    14      1   seq
....................uUaucucaguugguaauucaga......................................................................    1       1   seq
....................uaaucucaguugguaauCcaga......................................................................    23      1   seq
....................uaaucucaguuUguaauucaga......................................................................    34      1   seq
....................uaaucucaguugAuaauucaga......................................................................    23      1   seq
....................uaNucucaguugguaauucaga......................................................................    1       1   seq
....................Aaaucucaguugguaauucaga......................................................................    667     1   seq
....................uaaucucaguugguaUuucaga......................................................................    20      1   seq
....................uaaucCcaguugguaauucaga......................................................................    21      1   seq
....................uaauUucaguugguaauucaga......................................................................    9       1   seq
....................uaaucucaguugguaauucCga......................................................................    2       1   seq
....................uaaucucaUuugguaauucaga......................................................................    5       1   seq
....................uaaucucaguuggGaauucaga......................................................................    7       1   seq
....................uaaucucaguugguaaGucaga......................................................................    8       1   seq
....................uaaucucaguugguaauucagG......................................................................    196     1   seq
....................uaaucucaguugguaauucagU......................................................................    70      1   seq
....................uaaucucaguuCguaauucaga......................................................................    8       1   seq
....................uaaucucaguugguaauucaCa......................................................................    5       1   seq
....................uGaucucaguugguaauucaga......................................................................    59      1   seq
....................uaaucucaguugguaauuGaga......................................................................    7       1   seq
....................uaaucucaguugguaauGcaga......................................................................    4       1   seq
....................uaGucucaguugguaauucaga......................................................................    2129    1   seq
....................uaaucucaguugCuaauucaga......................................................................    18      1   seq
....................uaaAcucaguugguaauucaga......................................................................    87      1   seq
....................uaaucucaguugguaauuAaga......................................................................    91      1   seq
....................uaaucucaguGgguaauucaga......................................................................    7       1   seq
....................uaUucucaguugguaauucaga......................................................................    11      1   seq
....................uaaGcucaguugguaauucaga......................................................................    3       1   seq
....................uaaucAcaguugguaauucaga......................................................................    74      1   seq
....................uaaucucaguugguaaAucaga......................................................................    35      1   seq
....................uaaucuUaguugguaauucaga......................................................................    91      1   seq
....................uaaucucaguugguaauucGga......................................................................    47      1   seq
....................uaaucucaguugguCauucaga......................................................................    1       1   seq
....................uaaucucaguugguaauucaAa......................................................................    23      1   seq
....................uNaucucaguugguaauucaga......................................................................    5       1   seq
....................Gaaucucaguugguaauucaga......................................................................    71      1   seq
....................uaaucucaguAgguaauucaga......................................................................    80      1   seq
....................uaaucucaguugguaauucUga......................................................................    16      1   seq
....................Naaucucaguugguaauucaga......................................................................    57      1   seq
....................uaaucucaCuugguaauucaga......................................................................    7       1   seq
....................uaaucucagGugguaauucaga......................................................................    6       1   seq
....................uCaucucaguugguaauucagag.....................................................................    1       1   seq
....................uaaucuUaguugguaauucagag.....................................................................    53      1   seq
....................Gaaucucaguugguaauucagag.....................................................................    36      1   seq
....................uaaucucaguugguGauucagag.....................................................................    34      1   seq
....................uaCucucaguugguaauucagag.....................................................................    1       1   seq
....................uaaucucaguuCguaauucagag.....................................................................    3       1   seq
....................uaaucucaguuUguaauucagag.....................................................................    18      1   seq
....................uaaAcucaguugguaauucagag.....................................................................    43      1   seq
....................uaaucucaguugguaauucagaC.....................................................................    43      1   seq
....................uaaucucaguugguaauucGgag.....................................................................    18      1   seq
....................uaaucuGaguugguaauucagag.....................................................................    15      1   seq
....................uaaucucaguugguaauuUagag.....................................................................    40      1   seq
```

Mature **Star**

```
ccuccuagugacuaauugucuaaucucaguugguaauucagagugcuggaauucguucucggauuaacagcugggaucggacaaauaacacacuaacaucuacaccacaaaa
....................uaaucucCguugguaauucagag.....................................................................     1       1   seq
....................uaaucucaguugguaauucagag.....................................................................     73090   0   seq
....................uaaucucaguAgguaauucagag.....................................................................     28      1   seq
....................uaaucucagCugguaauucagag.....................................................................     6       1   seq
....................uaaucNcaguugguaauucagag.....................................................................     1       1   seq
....................uaaucucUguugguaauucagag.....................................................................     4       1   seq
....................uaaucucaguugguaauucagCg.....................................................................     1       1   seq
....................uaauAucaguugguaauucagag.....................................................................     15      1   seq
....................uaaGcucaguugguaauucagag.....................................................................     3       1   seq
....................uaaucucaguugguaaCucagag.....................................................................     15      1   seq
....................uaaucucaguugguaauucagaU.....................................................................     58      1   seq
....................uaaucCcaguugguaauucagag.....................................................................     9       1   seq
....................uaaucucaguugguaaGucagag.....................................................................     6       1   seq
....................uaaucucaAuugguaauucagag.....................................................................     4       1   seq
....................Naaucucaguugguaauucagag.....................................................................     19      1   seq
....................uaaucucagAugguaauucagag.....................................................................     32      1   seq
....................uaaucucaguugguaauucagGg.....................................................................     12      1   seq
....................uaaucAcaguugguaauucagag.....................................................................     36      1   seq
....................uaaucucaguCgguaauucagag.....................................................................     9       1   seq
....................uaGucucaguugguaauucagag.....................................................................     1103    1   seq
....................uaauGucaguugguaauucagag.....................................................................     7       1   seq
....................uaaucGcaguugguaauucagag.....................................................................     4       1   seq
....................uaaucucaguugUuaauucagag.....................................................................     2       1   seq
....................uaaucucaguugguaauucagUg.....................................................................     8       1   seq
....................uaaucucaguugguaauGcagag.....................................................................     3       1   seq
....................uaaucucaguugguaauAcagag.....................................................................     29      1   seq
....................uaUucucaguugguaauucagag.....................................................................     1       1   seq
....................uaaucucaguugguaauucaCag.....................................................................     2       1   seq
....................uaaucucaguugCuaauucagag.....................................................................     6       1   seq
....................uaaucucaguugguaauucCgag.....................................................................     2       1   seq
....................uaaucucaUuugguaauucagag.....................................................................     2       1   seq
....................uaaucucaguugguaUuucagag.....................................................................     8       1   seq
....................uaaucucaguugguaauucaAag.....................................................................     20      1   seq
....................Aaaucucaguugguaauucagag.....................................................................     294     1   seq
....................uaaucuAaguugguaauucagag.....................................................................     30      1   seq
....................uaaucucaguugguaauuGagag.....................................................................     6       1   seq
....................uaaucucaguuAguaauucagag.....................................................................     102     1   seq
....................Caaucucaguugguaauucagag.....................................................................     11      1   seq
....................uaaucucaguugguaauucagaN.....................................................................     1       1   seq
....................uaaucucaguugguaGuucagag.....................................................................     9       1   seq
....................uaaucucaguGgguaauucagag.....................................................................     3       1   seq
....................uaaucucaguugguaauucagaA.....................................................................     694     1   seq
....................uaaucucaguugguUauucagag.....................................................................     3       1   seq
....................uNaucucaguugguaauucagag.....................................................................     3       1   seq
....................uaaucucaguugguaauucUgag.....................................................................     14      1   seq
....................uaaucucaguugguaauCcagag.....................................................................     7       1   seq
....................uaauUucaguugguaauucagag.....................................................................     3       1   seq
....................uaaucucaguuggCaauucagag.....................................................................     6       1   seq
....................uaaucucagGugguaauucagag.....................................................................     3       1   seq
....................uaaucucaCuugguaauucagag.....................................................................     4       1   seq
....................uaaucucaguugguaauuAagag.....................................................................     35      1   seq
....................uaaucucGguugguaauucagag.....................................................................     19      1   seq
....................uaaucucaguugAuaauucagag.....................................................................     12      1   seq
....................uaaCcucaguugguaauucagag.....................................................................     7       1   seq
....................uaaucucaguuggAaauucagag.....................................................................     24      1   seq
....................uGaucucaguugguaauucagag.....................................................................     27      1   seq
....................uaaucucaguugguaaAucagag.....................................................................     17      1   seq
....................uaaucucaguugguaauucaUag.....................................................................     4       1   seq
....................uaaucucaguugguaauucagagA....................................................................     1586    1   seq
....................uaaucucaguAgguaauucagagu....................................................................     2       1   seq
....................uaaucucaguugguaUuucagagu....................................................................     1       1   seq
....................uaaucucaguugguaauucagagC....................................................................     130     1   seq
....................uaaucAcaguugguaauucagagu....................................................................     1       1   seq
....................uaaucucaguugguaauucagagN....................................................................     1       1   seq
....................Aaaucucaguugguaauucagagu....................................................................     1       1   seq
....................uaaucucaguugguaauucagaUu....................................................................     1       1   seq
....................uaaucuUaguugguaauucagagu....................................................................     1       1   seq
....................uaaucucaguugguaauucagaAu....................................................................     5       1   seq
....................uaGucucaguugguaauucagagu....................................................................     28      1   seq
....................uaaucucaguugguaauucagagG....................................................................     27      1   seq
```

```
                    Mature                                 Star
ccuccuagugacuaauugucuaaucucaguugguaauucagagugcuggaauucguucucggauuaacagcugggaucggacaaauaacacacuaacaucuacaccacaaaa
....................uaaucucaguugAuaauucagagu....................................................................    1          1          seq
....................uaaucucaguugguGauucagagu....................................................................    1          1          seq
....................uaaucucaguugguaauucagagu....................................................................    552        0          seq
....................uaaucucaguugguaauucaguU...................................................................    4          1          seq
....................uaaucucaguugguaauucagagugcugga..............................................................    88         0          seq
.....................aaucucaguugguaauuca........................................................................    8          0          seq
.....................aaucucaguugguaauucaA.......................................................................    1          1          seq
.....................aaucucaguugguaauucag.......................................................................    95         0          seq
.....................aaucucaguugguGauucag.......................................................................    1          1          seq
.....................Uaucucaguugguaauucag.......................................................................    1          1          seq
.....................aaucuUaguugguaauucaga......................................................................    2          1          seq
.....................Naucucaguugguaauucaga......................................................................    1          1          seq
.....................aaucucaguugguaUuucaga......................................................................    2          1          seq
.....................aaucucaguugUuaauucaga......................................................................    2          1          seq
.....................aaucucaguAgguaauucaga......................................................................    3          1          seq
.....................aaucucaguugguaauucagG......................................................................    8          1          seq
.....................aaucuAaguugguaauucaga......................................................................    1          1          seq
.....................aaucucaguugguaauuAaga......................................................................    1          1          seq
.....................Uaucucaguugguaauucaga......................................................................    10         1          seq
.....................aaucucaguugguaauuUaga......................................................................    1          1          seq
.....................aGucucaguugguaauucaga......................................................................    5          1          seq
.....................aaucucaguugguaauucagU......................................................................    6          1          seq
.....................aaucucaguugguaauucaga......................................................................    2387       0          seq
.....................aaucucagAugguaauucaga......................................................................    1          1          seq
.....................aaAcucaguugguaauucaga......................................................................    1          1          seq
.....................Gaucucaguugguaauucaga......................................................................    1          1          seq
.....................aaucucaguuAguaauucaga......................................................................    7          1          seq
.....................aaucucaguugguaauAcaga......................................................................    1          1          seq
.....................aaucucaguCgguaauucaga......................................................................    1          1          seq
.....................aaucucaguuggCaauucaga......................................................................    7          1          seq
.....................aaucucaguuggAaauucaga......................................................................    2          1          seq
.....................aaucucaguugguaauucGga......................................................................    3          1          seq
.....................aaucucaguugguaauucagaU.....................................................................    1          1          seq
.....................aaucucaguuUguaauucagag.....................................................................    2          1          seq
.....................aaucucaguugguaaAucagag.....................................................................    1          1          seq
.....................aaucuUaguugguaauucagag.....................................................................    1          1          seq
.....................aaucucaguugguGauucagag.....................................................................    1          1          seq
.....................aaucucGguugguaauucagag.....................................................................    1          1          seq
.....................aaucucaguugguaauuUagag.....................................................................    1          1          seq
.....................aaAcucaguugguaauucagag.....................................................................    1          1          seq
.....................aaucucaguuAguaauucagag.....................................................................    5          1          seq
.....................Naucucaguugguaauucagag.....................................................................    2          1          seq
.....................aaucucaguugguaauucagaA.....................................................................    16         1          seq
.....................aaucucaguuCguaauucagag.....................................................................    1          1          seq
.....................Uaucucaguugguaauucagag.....................................................................    2          1          seq
.....................aaCcucaguugguaauucagag.....................................................................    1          1          seq
.....................aaucucaguugguaauucagag.....................................................................    999        0          seq
.....................aauAucaguugguaauucagag.....................................................................    1          1          seq
.....................aaucucaguugguaauuAagag.....................................................................    1          1          seq
.....................aaucucaguugguaauucaAag.....................................................................    1          1          seq
.....................aauUucaguugguaauucagag.....................................................................    3          1          seq
.....................aaucucaguugguaauucagagG....................................................................    1          1          seq
.....................aGucucaguugguaauucagagu....................................................................    6          1          seq
.....................aaucucaguugguaaAucagagu....................................................................    1          1          seq
.....................aaAcucaguugguaauucagagu....................................................................    1          1          seq
.....................aaucucaguuAguaauucagagu....................................................................    1          1          seq
.....................aaucucaguugguaauucagagA....................................................................    37         1          seq
.....................aaucucaguugguaauucagagu....................................................................    345        0          seq
......................aucucaguugguaauucaga......................................................................    2          0          seq
......................aucucaguugguaauucagag.....................................................................    2          0          seq
......................aucucaguugguaauucagagu....................................................................    3          0          seq
......................aucucaguugguaauucagagug...................................................................    10         0          seq
.......................ucucaguugguaauucaga......................................................................    1          0          seq
.......................ucucaguugguaauucagag.....................................................................    1          0          seq
.......................ucucaguugguaauucagagug...................................................................    1          0          seq
.......................ucucaguugguaauucagagugc..................................................................    1          0          seq
........................cucaguugguaauucaga......................................................................    2          0          seq
........................cucaguugguaauucagag.....................................................................    2          0          seq
.........................ucaguugguaauucagag.....................................................................    1          0          seq
.........................ucaguugguaauucagagA....................................................................    1          1          seq
```

Mature Star

```
ccuccuagugacuaauugucuaaucucaguugguaauucagagugcuggaauucguucucggauuaacagcugggaucggacaaauaacacacuaacaucuacaccacaaaa
.....................................................cguucucggauuaacagcugggaucgga...............................	1	0	seq
........................................................ucucggauuaacagcugggauc..................................	1	0	seq
.........................................................cucggauuaacagcugggauc..................................	1	0	seq
.........................................................cucggauuaacagcugggaucU.................................	3	1	seq
..........................................................ucggauuaacagcuggga....................................	2	0	seq
..........................................................ucggauuaacagcugggau...................................	17	0	seq
..........................................................uUggauuaacagcugggauc..................................	1	1	seq
..........................................................ucggauuaacGgcugggauc..................................	1	1	seq
..........................................................ucggauuaacaCcugggauc..................................	1	1	seq
..........................................................Acggauuaacagcugggauc..................................	1	1	seq
..........................................................ucggauuGacagcugggauc..................................	1	1	seq
..........................................................ucggauuUacagcugggauc..................................	1	1	seq
..........................................................ucggauuaacagcugAgauc..................................	1	1	seq
..........................................................ucggauuaacagcugggauA..................................	5	1	seq
..........................................................ucggauuaacagcugggauc..................................	317	0	seq
..........................................................ucggauuaacagcugggaucg.................................	84	0	seq
..........................................................ucggauuaacagcugggauUg.................................	1	1	seq
..........................................................ucggauuaacagcugggaucU.................................	5	1	seq
..........................................................ucAgauuaacagcugggaucg.................................	1	1	seq
..........................................................ucggauuaacagcugggauUgg................................	1	1	seq
..........................................................ucggauGaacagcugggaucgg................................	1	1	seq
..........................................................ucAgauuaacagcugggaucgg................................	3	1	seq
..........................................................uAggauuaacagcugggaucgg................................	1	1	seq
..........................................................ucggauuaacagcugCgaucgg................................	1	1	seq
..........................................................uUggauuaacagcugggaucgg................................	3	1	seq
..........................................................ucggauuaacagcugggaucgg................................	727	0	seq
..........................................................ucggauCaacagcugggaucgg................................	1	1	seq
..........................................................ucggauuaacagcCgggaucgg................................	3	1	seq
..........................................................ucggauuaacagAugggaucgg................................	1	1	seq
..........................................................Acggauuaacagcugggaucgg................................	1	1	seq
..........................................................ucggauuaacagcugggaucgA................................	4	1	seq
..........................................................Gcggauuaacagcugggaucgg................................	1	1	seq
..........................................................ucggauuaacagcugggaAcgg................................	1	1	seq
..........................................................ucggauuaacagcuAggaucgg................................	2	1	seq
..........................................................ucggauuaacagcugggaucAga...............................	2	1	seq
..........................................................ucggauuaacagcugggauUgga...............................	1	1	seq
..........................................................ucggauCaacagcugggaucgga...............................	1	1	seq
..........................................................ucggauuaacagcugggauAgga...............................	1	1	seq
..........................................................ucggauuaacagcugggaucggC...............................	2	1	seq
..........................................................ucggauuaacagcugggaucggU...............................	424	1	seq
..........................................................Ccggauuaacagcugggaucgga...............................	1	1	seq
..........................................................Gcggauuaacagcugggaucgga...............................	3	1	seq
..........................................................ucggauuaUcagcugggaucgga...............................	1	1	seq
..........................................................ucggaAuaacagcugggaucgga...............................	1	1	seq
..........................................................uUggauuaacagcugggaucgga...............................	3	1	seq
..........................................................ucggauuaacagcugggaucgga...............................	500	0	seq
..........................................................ucggauuaacagcugggaucggG...............................	8	1	seq
..........................................................Acggauuaacagcugggaucgga...............................	1	1	seq
..........................................................ucggauuaacagcuAggaucgga...............................	2	1	seq
..........................................................ucggauuaacagcugggaucggaG..............................	1	1	seq
..........................................................ucggauuaacagcugggaucggaU..............................	17	1	seq
..........................................................ucggauuaacagcugggaucggaA..............................	8	1	seq
..........................................................ucggauuaacagcugggaucggaAa.............................	4	1	seq
..........................................................ucggauuaacagcugggaucggaAaaau..........................	3	1	seq
...........................................................cggauuaacagcugggau...................................	2	0	seq
...........................................................cggauuaacagcugggauc..................................	11	0	seq
...........................................................cggauuaacagcugggaucg.................................	3	0	seq
...........................................................cggauuaacagcuUggaucgg................................	1	1	seq
...........................................................cggauuaacagcugggauUgg................................	2	1	seq
...........................................................cggaAuaacagcugggaucgg................................	1	1	seq
...........................................................cggauuaacagcugggaucgU................................	2	1	seq
...........................................................cggauuaacagcugAgaucgg................................	1	1	seq
...........................................................cggauuaacagcugggaucgg................................	119	0	seq
...........................................................Nggauuaacagcugggaucgga...............................	1	1	seq
...........................................................cggaCuaacagcugggaucgga...............................	1	1	seq
...........................................................cggauuaacagcGgggaucgga...............................	1	1	seq
...........................................................cggauuaacagcuggCaucgga...............................	1	1	seq
...........................................................cggauuaacagcugggGucgga...............................	1	1	seq
...........................................................cgAauuaacagcugggaucgga...............................	1	1	seq
...........................................................cggauuaacagcugggaucgUa...............................	1	1	seq
```

```
                    Mature                  Star
ccuccuagugacuaauugucuaaucucaguugguaauucagagugcuggaauucguucucggauuaacagcugggaucggacaaauaacacacuaacaucuacaccacaaaa
...........................................................cggauuaacagcugAgaucgga...............................   3     1   seq
...........................................................cggauuaacagAugggaucgga...............................   1     1   seq
...........................................................cggauuaacagcugggaucAga...............................   10    1   seq
...........................................................cggauuaacagcugggaucggC...............................   2     1   seq
...........................................................cggaAuaacagcugggaucgga...............................   4     1   seq
...........................................................cggauGaacagcugggaucgga...............................   1     1   seq
...........................................................cggauuaacagcuggAaucgga...............................   1     1   seq
...........................................................cggauuaacagcugggauUgga...............................   7     1   seq
...........................................................cggauuaacagcugggaucgga...............................   2563  0   seq
...........................................................cggauuaacagcugggaucggG...............................   60    1   seq
...........................................................cggauuaacagcuAggaucgga...............................   5     1   seq
...........................................................cggauCaacagcugggaucgga...............................   2     1   seq
...........................................................cggauAaacagcugggaucgga...............................   1     1   seq
...........................................................cggauuaacagcugggaucggU...............................   5     1   seq
...........................................................cAgauuaacagcugggaucgga...............................   3     1   seq
...........................................................Uggauuaacagcugggaucgga...............................   2     1   seq
...........................................................cCgauuaacagcugggaucgga...............................   2     1   seq
...........................................................cggauuaacagcugggaucgAa...............................   1     1   seq
...........................................................cggauuaacagcugggUucgga...............................   1     1   seq
...........................................................cgCauuaacagcugggaucgga...............................   2     1   seq
...........................................................cggauuaaAagcugggaucgga...............................   1     1   seq
...........................................................Aggauuaacagcugggaucgga...............................   2     1   seq
...........................................................cggauuaacagcugggaucggac..............................   1     0   seq
...........................................................cggauuaacagcugggaucggaA..............................   24    1   seq
...........................................................cggauuaacagcugggaucggaU..............................   3     1   seq
............................................................ggauuaacagcugggaucggU...............................   1     1   seq
............................................................ggauuaacagcugggaucgga...............................   5     0   seq
.............................................................gauuaacagcugggaucggac..............................   1     0   seq
.............................................................gauuaacagcuAggaucggaca.............................   1     1   seq
..............................................................auuaacagcugggaucggac..............................   1     0   seq
..............................................................auuaacagcugggaucggaca.............................   10    0   seq
...............................................................uuaacagcugggaucggaca.............................   3     0   seq
...............................................................uuaacagcugggaucggacaaa...........................   11    0   seq
...............................................................uuaacagcugggaucggacaaau..........................   2     0   seq
...............................................................uuaacagcugggaucggacaaaA..........................   3     1   seq
.................................................................................caaauaacacacuaacaucuaca........   1     0   seq
```
